# Bis((*E*)-2-{3-[4-(1*H*-imidazol-1-yl-κ*N* ^3^)styr­yl]-5,5-di­methyl­cyclo­hex-2-enyl­idene}malono­nitrile)­diiodido­mercury(II)

**DOI:** 10.1107/S1600536813025191

**Published:** 2013-09-21

**Authors:** Wen-Gang Xi, Zhi-Chao Wu, Hong-Ping Zhou

**Affiliations:** a Department of Chemistry, Anhui University, Hefei 230039, People’s Republic of China; b Key Laboratory of Functional Inorganic Materials, Chemistry, Hefei 230039, People’s Republic of China

## Abstract

In the title compound, [HgI_2_(C_22_H_20_N_4_)_2_], the Hg^II^ cation is situated on a twofold rotation axis and is coordinated by two iodide anions and two imidazolyl N atoms in a distorted tetra­hedral geometry. In the crystal, C—H⋯I inter­actions link the mol­ecules into chains extending in [010], which are further linked into sheets parallel to (100) through C—H⋯N hydrogen bonding inter­actions.

## Related literature

For the crystal structure of the organic ligand of the title compound, see: Zheng *et al.* (2013 ▶). For mercury(II) complexes in which the Hg(II) cation is four-coordinated by two terminal iodide ions and two N atoms from organic ligands in a distorted tetra­hedral geometry, see: Li (2011 ▶); Shirvan *et al.* (2012 ▶).
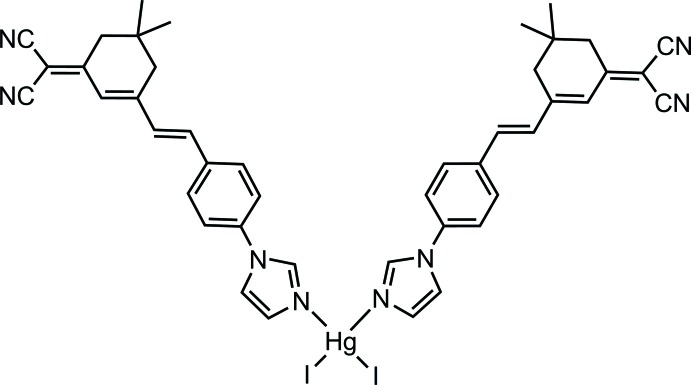


## Experimental

### 

#### Crystal data

[HgI_2_(C_22_H_20_N_4_)_2_]
*M* *_r_* = 1135.23
Monoclinic, 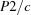

*a* = 18.768 (3) Å
*b* = 6.4890 (9) Å
*c* = 18.681 (3) Å
β = 103.896 (10)°
*V* = 2208.5 (5) Å^3^
*Z* = 2
Mo *K*α radiation
μ = 4.92 mm^−1^
*T* = 298 K
0.30 × 0.20 × 0.20 mm

#### Data collection

Bruker SMART APEX CCD diffractometer
Absorption correction: multi-scan (*SADABS*; Bruker, 2002 ▶) *T*_min_ = 0.320, *T*_max_ = 0.439
14665 measured reflections
3889 independent reflections
3647 reflections with *I* > 2σ(*I*)
*R*_int_ = 0.042

#### Refinement

*R*[*F* ^2^ > 2σ(*F* ^2^)] = 0.036
*wR*(*F* ^2^) = 0.092
*S* = 1.04
3889 reflections
251 parameters
H-atom parameters constrained
Δρ_max_ = 2.28 e Å^−3^
Δρ_min_ = −1.86 e Å^−3^

### 

Data collection: *SMART* (Bruker, 2002 ▶); cell refinement: *SAINT* (Bruker, 2002 ▶); data reduction: *SAINT*; program(s) used to solve structure: *SHELXS97* (Sheldrick, 2008 ▶); program(s) used to refine structure: *SHELXL97* (Sheldrick, 2008 ▶); molecular graphics: *SHELXTL* (Sheldrick, 2008 ▶); software used to prepare material for publication: *SHELXTL*.

## Supplementary Material

Crystal structure: contains datablock(s) I, Global. DOI: 10.1107/S1600536813025191/cq2006sup1.cif

Structure factors: contains datablock(s) I. DOI: 10.1107/S1600536813025191/cq2006Isup2.hkl

Additional supplementary materials:  crystallographic information; 3D view; checkCIF report

## Figures and Tables

**Table 1:** Hydrogen-bond geometry (Å, °)

*D*—H⋯*A*	*D*—H	H⋯*A*	*D*⋯*A*	*D*—H⋯*A*
C13—H13⋯N2^i^	0.93	2.70	3.541 (15)	151
C18—H18⋯I1^ii^	0.93	3.09	3.864 (5)	142

Symmetry codes: (i) 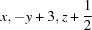; (ii) 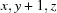.

**Fig. 1.**
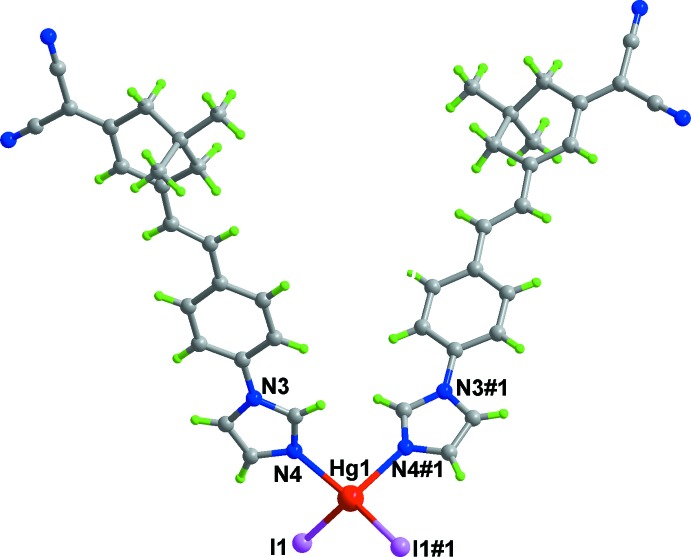
: The molecular structure of the title molecule. [Symmetry code: #1: -x + 1,y,-z + 3/2].

**Fig. 2.**
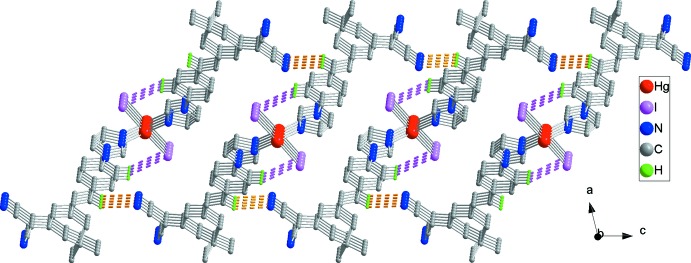
: Packing diagram of the title compound viewed along b axis. Intermolecular C—H···I and C—H···N interactions are shown as dashed lines in pink and yellow, respectively.

## supplementary crystallographic information

### 1. Comment

The organic ligand of the title compound has previously been investigated for its optical properties (Zheng *et al.*, 2013). In addition, some mercury(II) complexes in which the Hg(II) cation is four-coordinated by two terminal iodide ions and two nitrogen atoms from organic ligands to form distorted tetrahedral geometry have been reported (Li, 2011; Shirvan *et al.*, 2012). In this study, we report the crystal structure of the title compound (Fig. 1). In the molecular packing structure of the compound, intermolecular C—H···I interactions link the molecules into chains. The neighboring chains are further linked into sheets through C—H···N hydrogen bonding interactions (Fig.2). Intermolecular hydrogen bonds lengths and angles are reported in Table.1.

### 2. Experimental

For the preparation of the title compound, a solution of HgI_2_ (0.1 g, 0.22 mmol) in methanol (10 mL) was carefully layered on top of the surface of the solution of (*E*)-2-(3-(4-(1*H*-imidazol-1-yl)styryl)-5,5-dimethylcyclohex-2- enylidene) malononitrile (0.15 g, 0.44 mmol) in chloroform (10 mL). Crystals were obtained after a week at about 298 K (yield 0.16 g, 64.0%).

### 3. Refinement

All hydrogen atoms were placed in geometrically idealized positions and constrained to ride on their parent atoms, with C—H = 0.93 Å and *U*_iso_(H) = 1.2 *U*_eq_.

### Crystal data

**Table:**

[HgI_2_(C_22_H_20_N_4_)_2_]	*Z* = 2
*M**_r_* = 1135.23	*F*(000) = 1092
Monoclinic, *P*2/*c*	*D*_x_ = 1.707 Mg m^−^^3^
Hall symbol: -P 2yc	Mo *K*α radiation, λ = 0.71069 Å
*a* = 18.768 (3) Å	µ = 4.92 mm^−^^1^
*b* = 6.4890 (9) Å	*T* = 298 K
*c* = 18.681 (3) Å	Block, red
β = 103.896 (10)°	0.30 × 0.20 × 0.20 mm
*V* = 2208.5 (5) Å^3^	

### Data collection

**Table:**

Bruker SMART APEX CCD diffractometer	3889 independent reflections
Radiation source: fine-focus sealed tube	3647 reflections with *I* > 2σ(*I*)
Graphite monochromator	*R*_int_ = 0.042
phi and ω scans	θ_max_ = 25.0°, θ_min_ = 2.2°
Absorption correction: multi-scan (*SADABS*; Bruker, 2002)	*h* = −21→22
*T*_min_ = 0.320, *T*_max_ = 0.439	*k* = −7→7
14665 measured reflections	*l* = −22→22

### Refinement

**Table:**

Refinement on *F*^2^	Primary atom site location: structure-invariant direct methods
Least-squares matrix: full	Secondary atom site location: difference Fourier map
*R*[*F*^2^ > 2σ(*F*^2^)] = 0.036	Hydrogen site location: inferred from neighbouring sites
*wR*(*F*^2^) = 0.092	H-atom parameters constrained
*S* = 1.04	*w* = 1/[σ^2^(*F*_o_^2^) + (0.0496*P*)^2^ + 4.7641*P*] where *P* = (*F*_o_^2^ + 2*F*_c_^2^)/3
3889 reflections	(Δ/σ)_max_ = 0.001
251 parameters	Δρ_max_ = 2.28 e Å^−^^3^
0 restraints	Δρ_min_ = −1.86 e Å^−^^3^

### Special details

**Table:**

Geometry. All e.s.d.'s (except the e.s.d. in the dihedral angle between two l.s. planes) are estimated using the full covariance matrix. The cell e.s.d.'s are taken into account individually in the estimation of e.s.d.'s in distances, angles and torsion angles; correlations between e.s.d.'s in cell parameters are only used when they are defined by crystal symmetry. An approximate (isotropic) treatment of cell e.s.d.'s is used for estimating e.s.d.'s involving l.s. planes.
Refinement. Refinement of *F*^2^ against ALL reflections. The weighted *R*-factor *wR* and goodness of fit *S* are based on *F*^2^, conventional *R*-factors *R* are based on *F*, with *F* set to zero for negative *F*^2^. The threshold expression of *F*^2^ > σ(*F*^2^) is used only for calculating *R*-factors(gt) *etc*. and is not relevant to the choice of reflections for refinement. *R*-factors based on *F*^2^ are statistically about twice as large as those based on *F*, and *R*- factors based on ALL data will be even larger.

### Fractional atomic coordinates and isotropic or equivalent isotropic displacement parameters (Å^2^)

**Table:**

	*x*	*y*	*z*	*U*_iso_*/*U*_eq_	
Hg1	0.5000	−0.05717 (4)	0.7500	0.04225 (11)	
I1	0.40548 (2)	−0.27343 (6)	0.811929 (18)	0.05948 (14)	
N1	0.0943 (6)	2.1499 (14)	0.1997 (5)	0.136 (4)	
N2	0.2337 (6)	1.6553 (14)	0.1582 (5)	0.125 (3)	
N3	0.4072 (2)	0.4228 (6)	0.5797 (2)	0.0361 (8)	
N4	0.4591 (2)	0.1711 (6)	0.6528 (2)	0.0393 (9)	
C1	0.2004 (5)	1.7214 (12)	0.1961 (4)	0.083 (2)	
C2	0.1228 (5)	1.9987 (13)	0.2193 (5)	0.081 (2)	
C3	0.1591 (3)	1.8091 (9)	0.2440 (4)	0.0589 (15)	
C4	0.1556 (3)	1.7198 (8)	0.3090 (3)	0.0465 (12)	
C5	0.1146 (3)	1.8174 (9)	0.3589 (3)	0.0541 (13)	
H5A	0.0727	1.8906	0.3293	0.065*	
H5B	0.1461	1.9179	0.3896	0.065*	
C6	0.0875 (3)	1.6647 (9)	0.4086 (3)	0.0521 (13)	
C7	0.0272 (4)	1.5276 (13)	0.3621 (4)	0.0748 (19)	
H7A	0.0469	1.4494	0.3277	0.112*	
H7B	−0.0124	1.6123	0.3356	0.112*	
H7C	0.0092	1.4352	0.3939	0.112*	
C8	0.0573 (5)	1.7859 (13)	0.4649 (4)	0.088 (2)	
H8A	0.0171	1.8704	0.4396	0.132*	
H8B	0.0953	1.8718	0.4935	0.132*	
H8C	0.0406	1.6918	0.4969	0.132*	
C9	0.1524 (3)	1.5320 (8)	0.4483 (3)	0.0501 (13)	
H9A	0.1849	1.6155	0.4853	0.060*	
H9B	0.1341	1.4208	0.4736	0.060*	
C10	0.1955 (3)	1.4417 (7)	0.3981 (3)	0.0419 (11)	
C11	0.1948 (3)	1.5315 (8)	0.3326 (3)	0.0474 (12)	
H11	0.2206	1.4693	0.3017	0.057*	
C12	0.2392 (3)	1.2574 (8)	0.4195 (3)	0.0471 (12)	
H12	0.2619	1.2009	0.3849	0.057*	
C13	0.2497 (3)	1.1623 (8)	0.4841 (3)	0.0454 (11)	
H13	0.2279	1.2220	0.5188	0.055*	
C14	0.2919 (3)	0.9732 (7)	0.5066 (3)	0.0421 (11)	
C15	0.3278 (3)	0.8633 (8)	0.4615 (3)	0.0487 (12)	
H15	0.3264	0.9124	0.4145	0.058*	
C16	0.3653 (3)	0.6840 (8)	0.4851 (3)	0.0461 (12)	
H16	0.3890	0.6139	0.4540	0.055*	
C17	0.3677 (3)	0.6079 (7)	0.5546 (2)	0.0366 (10)	
C18	0.3317 (3)	0.7111 (8)	0.6003 (3)	0.0469 (12)	
H18	0.3324	0.6597	0.6470	0.056*	
C19	0.2945 (3)	0.8915 (8)	0.5759 (3)	0.0485 (12)	
H19	0.2705	0.9605	0.6069	0.058*	
C20	0.4439 (3)	0.3009 (9)	0.5405 (3)	0.0488 (12)	
H20	0.4467	0.3207	0.4920	0.059*	
C21	0.4750 (3)	0.1469 (8)	0.5857 (3)	0.0476 (12)	
H21	0.5030	0.0407	0.5732	0.057*	
C22	0.4183 (3)	0.3371 (7)	0.6475 (3)	0.0413 (11)	
H22	0.3995	0.3893	0.6856	0.050*	

### Atomic displacement parameters (Å^2^)

**Table:**

	*U*^11^	*U*^22^	*U*^33^	*U*^12^	*U*^13^	*U*^23^
Hg1	0.0579 (2)	0.04093 (17)	0.03286 (16)	0.000	0.02053 (12)	0.000
I1	0.0757 (3)	0.0706 (3)	0.0394 (2)	−0.0295 (2)	0.02794 (18)	−0.01058 (15)
N1	0.165 (8)	0.101 (6)	0.144 (8)	0.062 (6)	0.038 (6)	0.065 (6)
N2	0.177 (8)	0.111 (6)	0.123 (6)	0.046 (6)	0.104 (7)	0.048 (5)
N3	0.040 (2)	0.037 (2)	0.0310 (19)	−0.0014 (16)	0.0078 (16)	0.0001 (15)
N4	0.054 (2)	0.035 (2)	0.0290 (18)	0.0050 (18)	0.0109 (17)	0.0047 (15)
C1	0.102 (6)	0.079 (5)	0.079 (5)	0.022 (4)	0.046 (5)	0.039 (4)
C2	0.094 (6)	0.073 (4)	0.078 (5)	0.021 (4)	0.021 (4)	0.030 (4)
C3	0.056 (3)	0.055 (3)	0.068 (4)	0.009 (3)	0.018 (3)	0.021 (3)
C4	0.039 (3)	0.044 (3)	0.054 (3)	0.001 (2)	0.008 (2)	0.008 (2)
C5	0.058 (3)	0.042 (3)	0.062 (3)	0.009 (2)	0.014 (3)	0.004 (2)
C6	0.049 (3)	0.055 (3)	0.054 (3)	0.005 (3)	0.017 (2)	−0.001 (3)
C7	0.048 (4)	0.090 (5)	0.085 (5)	−0.011 (3)	0.014 (3)	0.004 (4)
C8	0.105 (6)	0.091 (5)	0.081 (5)	0.036 (5)	0.047 (5)	0.005 (4)
C9	0.058 (3)	0.045 (3)	0.047 (3)	−0.001 (2)	0.013 (3)	0.001 (2)
C10	0.038 (3)	0.037 (3)	0.048 (3)	−0.0038 (19)	0.006 (2)	0.002 (2)
C11	0.049 (3)	0.043 (3)	0.053 (3)	0.003 (2)	0.018 (2)	0.008 (2)
C12	0.050 (3)	0.041 (3)	0.053 (3)	0.001 (2)	0.018 (2)	0.005 (2)
C13	0.050 (3)	0.040 (3)	0.046 (3)	0.000 (2)	0.010 (2)	−0.002 (2)
C14	0.046 (3)	0.037 (2)	0.041 (3)	−0.002 (2)	0.007 (2)	0.002 (2)
C15	0.060 (3)	0.046 (3)	0.043 (3)	0.003 (3)	0.019 (2)	0.012 (2)
C16	0.055 (3)	0.046 (3)	0.043 (3)	0.007 (2)	0.023 (2)	0.007 (2)
C17	0.041 (3)	0.033 (2)	0.033 (2)	−0.002 (2)	0.0049 (19)	−0.0004 (18)
C18	0.062 (3)	0.048 (3)	0.030 (2)	0.006 (2)	0.011 (2)	0.003 (2)
C19	0.059 (3)	0.048 (3)	0.039 (3)	0.012 (3)	0.013 (2)	−0.002 (2)
C20	0.063 (3)	0.055 (3)	0.033 (2)	0.013 (3)	0.020 (2)	0.006 (2)
C21	0.061 (3)	0.049 (3)	0.037 (2)	0.015 (3)	0.019 (2)	0.002 (2)
C22	0.054 (3)	0.040 (3)	0.034 (2)	0.002 (2)	0.018 (2)	0.0008 (19)

### Geometric parameters (Å, º)

**Table:**

Hg1—N4^i^	2.326 (4)	C8—H8B	0.9600
Hg1—N4	2.326 (4)	C8—H8C	0.9600
Hg1—I1^i^	2.7277 (4)	C9—C10	1.499 (8)
Hg1—I1	2.7277 (4)	C9—H9A	0.9700
N1—C2	1.135 (10)	C9—H9B	0.9700
N2—C1	1.135 (10)	C10—C11	1.351 (7)
N3—C22	1.353 (6)	C10—C12	1.451 (7)
N3—C20	1.371 (6)	C11—H11	0.9300
N3—C17	1.430 (6)	C12—C13	1.328 (7)
N4—C22	1.311 (6)	C12—H12	0.9300
N4—C21	1.366 (6)	C13—C14	1.466 (7)
C1—C3	1.435 (10)	C13—H13	0.9300
C2—C3	1.429 (9)	C14—C19	1.389 (7)
C3—C4	1.362 (8)	C14—C15	1.394 (8)
C4—C11	1.440 (7)	C15—C16	1.375 (7)
C4—C5	1.486 (8)	C15—H15	0.9300
C5—C6	1.525 (8)	C16—C17	1.380 (7)
C5—H5A	0.9700	C16—H16	0.9300
C5—H5B	0.9700	C17—C18	1.384 (7)
C6—C8	1.527 (9)	C18—C19	1.383 (7)
C6—C9	1.529 (8)	C18—H18	0.9300
C6—C7	1.535 (9)	C19—H19	0.9300
C7—H7A	0.9600	C20—C21	1.348 (7)
C7—H7B	0.9600	C20—H20	0.9300
C7—H7C	0.9600	C21—H21	0.9300
C8—H8A	0.9600	C22—H22	0.9300
			
N4^i^—Hg1—N4	100.90 (19)	C10—C9—H9A	108.8
N4^i^—Hg1—I1^i^	122.15 (10)	C6—C9—H9A	108.8
N4—Hg1—I1^i^	97.07 (10)	C10—C9—H9B	108.8
N4^i^—Hg1—I1	97.07 (10)	C6—C9—H9B	108.8
N4—Hg1—I1	122.15 (10)	H9A—C9—H9B	107.7
I1^i^—Hg1—I1	118.08 (2)	C11—C10—C12	119.1 (5)
C22—N3—C20	106.1 (4)	C11—C10—C9	120.6 (5)
C22—N3—C17	127.2 (4)	C12—C10—C9	120.3 (5)
C20—N3—C17	126.6 (4)	C10—C11—C4	122.4 (5)
C22—N4—C21	106.1 (4)	C10—C11—H11	118.8
C22—N4—Hg1	131.2 (3)	C4—C11—H11	118.8
C21—N4—Hg1	122.6 (3)	C13—C12—C10	125.7 (5)
N2—C1—C3	178.8 (10)	C13—C12—H12	117.1
N1—C2—C3	179.6 (10)	C10—C12—H12	117.1
C4—C3—C2	122.2 (6)	C12—C13—C14	127.0 (5)
C4—C3—C1	122.5 (5)	C12—C13—H13	116.5
C2—C3—C1	115.3 (6)	C14—C13—H13	116.5
C3—C4—C11	120.2 (5)	C19—C14—C15	117.0 (5)
C3—C4—C5	121.4 (5)	C19—C14—C13	118.8 (5)
C11—C4—C5	118.3 (5)	C15—C14—C13	124.1 (5)
C4—C5—C6	113.7 (5)	C16—C15—C14	121.6 (5)
C4—C5—H5A	108.8	C16—C15—H15	119.2
C6—C5—H5A	108.8	C14—C15—H15	119.2
C4—C5—H5B	108.8	C15—C16—C17	120.2 (5)
C6—C5—H5B	108.8	C15—C16—H16	119.9
H5A—C5—H5B	107.7	C17—C16—H16	119.9
C5—C6—C8	108.5 (5)	C16—C17—C18	119.8 (5)
C5—C6—C9	108.6 (5)	C16—C17—N3	120.5 (4)
C8—C6—C9	109.8 (5)	C18—C17—N3	119.7 (4)
C5—C6—C7	109.9 (5)	C19—C18—C17	119.2 (5)
C8—C6—C7	110.0 (6)	C19—C18—H18	120.4
C9—C6—C7	110.0 (5)	C17—C18—H18	120.4
C6—C7—H7A	109.5	C18—C19—C14	122.2 (5)
C6—C7—H7B	109.5	C18—C19—H19	118.9
H7A—C7—H7B	109.5	C14—C19—H19	118.9
C6—C7—H7C	109.5	C21—C20—N3	106.9 (4)
H7A—C7—H7C	109.5	C21—C20—H20	126.5
H7B—C7—H7C	109.5	N3—C20—H20	126.5
C6—C8—H8A	109.5	C20—C21—N4	109.4 (4)
C6—C8—H8B	109.5	C20—C21—H21	125.3
H8A—C8—H8B	109.5	N4—C21—H21	125.3
C6—C8—H8C	109.5	N4—C22—N3	111.4 (4)
H8A—C8—H8C	109.5	N4—C22—H22	124.3
H8B—C8—H8C	109.5	N3—C22—H22	124.3
C10—C9—C6	113.8 (5)		
			
N4^i^—Hg1—N4—C22	−46.1 (4)	C11—C10—C12—C13	174.5 (5)
I1^i^—Hg1—N4—C22	−170.9 (4)	C9—C10—C12—C13	−5.0 (8)
I1—Hg1—N4—C22	59.5 (5)	C10—C12—C13—C14	178.3 (5)
N4^i^—Hg1—N4—C21	134.9 (4)	C12—C13—C14—C19	−177.6 (5)
I1^i^—Hg1—N4—C21	10.1 (4)	C12—C13—C14—C15	−0.3 (9)
I1—Hg1—N4—C21	−119.5 (4)	C19—C14—C15—C16	−1.1 (8)
N1—C2—C3—C4	113 (100)	C13—C14—C15—C16	−178.4 (5)
N1—C2—C3—C1	−67 (100)	C14—C15—C16—C17	0.3 (8)
N2—C1—C3—C4	−128 (47)	C15—C16—C17—C18	0.7 (8)
N2—C1—C3—C2	51 (48)	C15—C16—C17—N3	−179.3 (5)
C2—C3—C4—C11	−178.3 (6)	C22—N3—C17—C16	175.5 (5)
C1—C3—C4—C11	0.8 (10)	C20—N3—C17—C16	−1.7 (8)
C2—C3—C4—C5	−1.2 (10)	C22—N3—C17—C18	−4.6 (7)
C1—C3—C4—C5	177.9 (7)	C20—N3—C17—C18	178.2 (5)
C3—C4—C5—C6	154.3 (6)	C16—C17—C18—C19	−1.0 (8)
C11—C4—C5—C6	−28.5 (7)	N3—C17—C18—C19	179.1 (5)
C4—C5—C6—C8	171.2 (6)	C17—C18—C19—C14	0.1 (9)
C4—C5—C6—C9	51.9 (6)	C15—C14—C19—C18	0.9 (8)
C4—C5—C6—C7	−68.5 (7)	C13—C14—C19—C18	178.4 (5)
C5—C6—C9—C10	−49.4 (6)	C22—N3—C20—C21	0.4 (6)
C8—C6—C9—C10	−167.9 (5)	C17—N3—C20—C21	178.1 (5)
C7—C6—C9—C10	70.9 (6)	N3—C20—C21—N4	−0.5 (7)
C6—C9—C10—C11	23.9 (7)	C22—N4—C21—C20	0.4 (6)
C6—C9—C10—C12	−156.7 (5)	Hg1—N4—C21—C20	179.7 (4)
C12—C10—C11—C4	−177.0 (5)	C21—N4—C22—N3	−0.2 (6)
C9—C10—C11—C4	2.4 (8)	Hg1—N4—C22—N3	−179.3 (3)
C3—C4—C11—C10	177.1 (6)	C20—N3—C22—N4	−0.2 (6)
C5—C4—C11—C10	−0.1 (8)	C17—N3—C22—N4	−177.8 (4)

Symmetry code: (i) −*x*+1, *y*, −*z*+3/2.

### Hydrogen-bond geometry (Å, º)

**Table:**

*D*—H···*A*	*D*—H	H···*A*	*D*···*A*	*D*—H···*A*
C13—H13···N2^ii^	0.93	2.70	3.541 (15)	151
C18—H18···I1^iii^	0.93	3.09	3.864 (5)	142

Symmetry codes: (ii) *x*, −*y*+3, *z*+1/2; (iii) *x*, *y*+1, *z*.
